# Clinical characteristics of sepsis-induced acute kidney injury in patients undergoing continuous renal replacement therapy

**DOI:** 10.1080/0886022X.2018.1489288

**Published:** 2018-07-17

**Authors:** A. Young Cho, Hyun Ju Yoon, Kwang Young Lee, In O. Sun

**Affiliations:** Department of Internal Medicine, Division of Nephrology, Presbyterian Medical Center, Jeonju, Korea

**Keywords:** Acute kidney injury, continuous renal replacement therapy, sepsis, platelet, urine output

## Abstract

**Objective**: The aim of this study was to investigate the clinical characteristics of sepsis-induced acute kidney injury (AKI) in patients undergoing continuous renal replacement therapy (CRRT).

**Methods**: From 2011 to 2015, we enrolled 340 patients who were treated with CRRT for sepsis at the Presbyterian Medical Center. In all patients, CRRT was performed using the PRISMA platform. We divided these patients into two groups (survivors and non-survivors) according to the 28-day all-cause mortality. We compared clinical characteristics and analyzed the predictors of mortality.

**Results:** The 28-day all-cause mortality was 62%. Survivors were younger than non-survivors and had higher platelet counts (178 ± 101 × 10^3^/mL vs. 134 ± 84 × 10^3^/mL, *p* < .01) and serum creatinine levels (4.2 ± 2.8 vs. 3.3 ± 2.7, *p* < .01). However, survivors had lower red blood cell distribution width (RDW) scores (14.9 ± 2.1 vs. 16.1 ± 3.3, *p* < .01) and APACHE II scores (24.5 ± 5.8 vs. 26.9 ± 5.7, *p* < .01) than non-survivors. Furthermore, survivors were more likely than non-survivors to have a urine output of >0.05 mL/kg/h (66% vs. 86%, *p* = .001) in the first day. In a multivariate logistic regression analysis, age, platelet count, RDW score, APACHE II score, serum creatinine level, and a urine output of <0.05 mL/kg/h the first day were prognostic factors for the 28-day all-cause mortality.

**Conclusion:** Age, platelet count, APACHE II score, RDW score, serum creatinine level, and urine output the first day are useful predictors for the 28-day all-cause mortality in sepsis patients requiring CRRT.

## Introduction

Acute kidney injury (AKI) is a common and serious complication in critically ill patients, and the development of AKI increases morbidity and mortality in these patients [1–4]. Sepsis is the leading cause of AKI treated with renal replacement therapy (RRT) in critically ill patients. The overall incidence of sepsis-induced AKI among all intensive care unit (ICU) admissions typically ranges between 15 and 20% [5].

Continuous renal replacement therapy (CRRT) is the established treatment modality in critically ill patients with AKI in the ICU [6]. Several predictors of survival in patients on CRRT have been described based on cross-sectional and retrospective studies [7–9]. It has been reported that sepsis-induced AKI is distinct from AKI in its pathophysiology without sepsis [10–12]. Therefore, sepsis-induced AKI may be distinguished from non-septic AKI in patient characteristics and clinical outcomes. Septic AKI patients have a poor prognosis compared to non-septic patients [13]. However, there have been few studies about the prognostic factors predicting mortality in patients with sepsis-induced AKI undergoing CRRT. Therefore, we investigated the clinical characteristics of sepsis-induced AKI patients undergoing CRRT and evaluated the predictors of mortality.

## Methods

### Patient selection

From 2011 to 2015, all 340 enrolled patients were ≥18 years, suffered from sepsis and AKI in the Presbyterian Medical Center ICU, required CRRT, and met at least one of the following criteria: oliguria (urine output <100 mL in a 6-h period and unresponsive to fluid resuscitation), serum potassium concentration >6.5 mmol/L, severe acidemia (pH <7.2), or presence of severe organ edema (e.g., pulmonary edema). Sepsis is defined as the presence (probable or documented) of infection together with systemic manifestations of infection. We defined severe sepsis as sepsis associated with acute organ dysfunction and septic shock as sepsis with acute circulatory failure characterized by persistent arterial hypotension (systolic arterial pressure <90 mmHg, mean arterial pressure <60 mmHg, or a reduction in systolic blood pressure >40 mmHg from baseline) despite adequate volume resuscitation [14]. We excluded 15 patients who died within the first 24 h of CRRT treatment, who were <18 years of age, who were on chronic dialysis, or who were diagnosed with terminal malignancy. We divided patients into two groups (survivors and non-survivors) according to the 28-day all-cause mortality, compared their clinical characteristics, and analyzed the predictors of survival. This study was approved by the Institutional Review Board of the Presbyterian Medical Center (Jeonju, South Korea).

### Clinical and laboratory information

All patients underwent detailed clinical history-taking, an examination, and a standard set of investigations including complete blood count, chest radiograph, urinalysis, two blood cultures, and tests for liver function, serum creatinine, urea, and electrolytes. The AKI was defined according to the Kidney Disease: Improving Global Outcome (KDIGO) guidelines [15]. Acute Physiology and Chronic Health Evaluation (APACHE) II and Sepsis-related organ failure assessment (SOFA) scores for all patients were obtained at the initial time of CRRT. The reference range for red blood cell distribution width (RDW) levels in this study was 12.2–14.8%. The patients’ weights were obtained in a variety of setting: the emergency room, nursing floor, and the ICU. Standard hospital scales were used for ambulatory patients and bed scales for non-ambulatory or ICU patients. The first available documented weight on the hospital record was taken as the initial weight, the majority of which were in the ICU. Subsequent daily weight was monitored with bed scales by the nursing staff and recoded on the care flow sheets. In all patients, continuous venovenous hemodiafiltration (CVVHDF) was performed using the PRISMA platform (Gambro, Hechingen, Germany). The CRRT prescriptions are shown in Table 1. Recovery of renal function was classified as complete when eGFR was within 25% of the reference eGFR (based on reference serum creatinine). Incomplete kidney recovery was defined as patients who had a 25% or greater decline of reference eGFR.

**Table 1.** CRRT Protocol.

Venous access	Femoral vein or internal jugular vein
CRRT mode	Continuous venovenous hemofiltration using the PRISMA platform (Gambro, Hechingen, Germany)
CRRT dose	40 mL/kg/h
CRRT blood flow	Initiation with blood flow of 100 mL/min, which was gradually increased to a rate of 150 mL/min
Replacement fluid	Commercially prepared, lactate-free, bicarbonate-buffered fluid (Hemosol; Gambro) was used as dialysate and replacement solution. The replacement fluid and dialysate flow rate were set as 1000 ml/h. Replacement fluid was infused in predilution mode.
Net ultrafiltration rate	Ultrafiltration rate was individualized.
Anticoagulation	Filters were primed with 2L of isotonic saline containing 10 000 U/l of heparin, and then 1 U/kg to 20 U/kg per hour of heparin was given. The goal of heparinization was to maintain systemic prefilter activated partial thromboplastin time between 45 and 55 seconds (1.5 × control) as described in a previous report [49]. In case of patients with bleeding risk, filters were primed with 2L of isotonic saline containing 50 mg/l of nafamostat mesilate, and then 10 mg/h to 30 mg/h was given.

### Statistical analysis

All data are presented as means ± standard deviations unless otherwise specified. The baseline characteristics of patients in the survivor and non-survivor groups were compared using *t-*tests, chi-square tests, or Fisher’s exact tests, as appropriate. A multiple logistic regression analysis was applied to predict the 28-day all-cause mortality after starting CRRT. A *p*-value of < .05 was considered to be statistically significant. All statistical analyses were carried out using SPSS version 22.0 (SPSS Inc., Chicago, IL).

## Results

### Baseline characteristics

The baseline characteristics of the 340 study subjects are presented in Table 2. The patients included 214 (63%) men and 126 (37%) women, with a mean age of 67 years (range, 21–92). The initial hemoglobin and mean RDW levels were 10.4 g/dL and 15.7%, respectively. The mean creatinine level at the start of CRRT was 3.6 mg/dL, and the mean APACHE II score was 26.0 (range, 7–42). The mean platelet count was 178 × 10^3^/mL. The most common cause of sepsis was pneumonia. The overall 28-day mortality was 62% (212/340). Of 128 survivors, 66% had complete recovery of renal function, 22% had incomplete recovery, and 12% had end-stage renal disease requiring RRT.

### Comparison of clinical characteristics between survivors and non-survivors

When we compared clinical characteristics between survivors (*n* = 212) and non-survivors (*n* = 128), the survivors were younger than non-survivors (64 ± 14 years vs. 69 ± 12 years, *p* < .01) and had higher serum creatinine levels (4.2 ± 2.8 vs. 3.3 ± 2.7, *p* < .01) and platelet counts (178 ± 101 vs. 134 ± 84, *p* < .01). However, survivors had lower RDW values (14.9 ± 2.1 vs. 16.1 ± 3.3, *p* < .01) and lower APACHE II scores (24.5 ± 5.8 vs. 26.9 ± 5.7, *p* < .01) than non-survivors. Survivors were more likely than non-survivors (82% vs. 66%, *p* < .01) to have a urine output of >0.05 mL/kg/h (66% vs. 86%, *p* = .001) during the first day. Non-survivors had more liver cirrhosis (18% vs. 7%, *p* = .003) and lower serum albumin levels (2.8 ± 0.6 vs. 3.0 ± 0.7, *p* < .01) than survivors (Table 3).

**Table 2.** The clinical and laboratory findings of the 340 patients with sepsis-induced AKI undergoing CRRT.

Characteristics	
Age, years	68 ± 13
Male, *n* (%)	214 (63)
Sepsis status	
Sepsis, *n* (%)	10 (3.0)
Severe sepsis, *n* (%)	89 (26.3)
Septic shock, *n* (%)	241 (70.7)
Cause of sepsis	
Thoracic, *n* (%)	205 (60.3)
Intra-abdominal, *n* (%)	45 (13.2)
Urogenital, *n* (%)	34 (10)
Skin/soft tissue/bone, *n* (%)	11 (3.2)
Other, *n* (%)	19 (5.6)
Source unknown, *n* (%)	26 (7.7)
Indication of CRRT	
Oliguria, *n* (%)	37 (10.9)
Hyperkalemia, *n* (%)	10 (2.9)
Acidemia, *n* (%)	31 (9.1)
Organ edema, *n* (%)	41 (12.1)
Mixed, *n* (%)	221 (65)
Comorbidity, *n* (%)	
Diabetes, *n* (%)	157 (46)
Hypertension, *n* (%)	225 (66)
Congestive heart failure, *n* (%)	77 (23)
Liver cirrhosis, *n* (%)	47 (14)
Chronic kidney disease, *n* (%)	114 (33)
Ischemic heart disease, *n* (%)	108 (32)
Systolic blood pressure (mmHg)	82 ± 23
Diastolic blood pressure (mmHg)	51 ± 14
APACHE2 score	26.0 ± 5.9
SOFA score	11.7 ± 3.2
RBC transfusion, unit	2.8 ± 4.1
Hemoglobin (mg/dl)	10.4 ± 2.4
Serum creatinine (mg/dl)	3.6 ± 2.8
Total bilirubin (mg/dl)	2.1 ± 5.0
Serum albumin (mg/dl)	2.9 ± 0.6
WBC count (× 10^3^/ mL)	15.1 ± 9.2
C-reactive protein (mg/dl)	11.5 ± 11.0
RDW (%)	15.7 ± 2.9
Electrolyte	
Na (mmol/L)	137 ± 10
K (mmol/L)	4.4 ± 1.5
Cl (mmol/L)	101 ± 8
Ca (mg/dl)	7.9 ± 0.9
P (mg/dl)	5.0 ± 2.8
Mg (mmol/L)	2.4 ± 0.6
ABGA	
pH	7.28 ± 0.16
PaO2 (mmHg)	97.4 ± 49.7
PaCO2 (mmHg)	33.5 ± 13.0
HCO3 (mmol/L)	14.6 ± 6.8
Lactic acid level^a^ (mmol/L)	7.6 ± 7.2
Platelet count (× 10^3^/ mL)	150 ± 93
UO <0.5 ml/kg/h during D1, *n* (%)	266 (78)
Renal outcome	
Complete recovery, *n* (%)	84 (66)
Incomplete recovery, *n* (%)	28 (22)
Dialysis dependence, *n* (%)	16 (12)
Death, *n* (%)	212 (62)

APACHE: Acute Physiology and Chronic Health Evaluation; SOFA: sepsis-related organ failure; RBC: red blood cell; WBC: white blood cell; RDW: red blood cell distribution width; UO: urine output; ABGA: arterial blood gas analysis.

a Lactic acid level was available in 255 patients.

**Table 3.** Comparison of baseline characteristics between survivors and non-survivors.

	Survivor (*n* = 128)	Non-survivor (*n* = 212)	*p*-value
Age	64 ± 14	69 ± 12	.001
Male, *n* (%)	79 (40)	135 (63)	NS
Sepsis status			
Sepsis, *n* (%)	5	5	NS
Severe sepsis, *n* (%)	40	49	NS
Septic shock, *n* (%)	71	170	<.001
Cause of sepsis			
Thoracic, *n* (%)	98 (48)	107 (52)	NS
Intra-abdominal, *n* (%)	21 (47)	24 (53)	NS
Urogenital, *n* (%)	14 (41)	20 (59)	NS
Skin/soft tissue/bone, *n* (%)	6 (55)	5 (45)	NS
Other, *n* (%)	11 (58)	8 (42)	NS
Source unknown, *n* (%)	11 (42)	15 (58)	NS
Indication of CRRT			
Oliguria, *n* (%)	17 (46)	20 (54)	NS
Hyperkalemia, *n* (%)	4 (40)	6 (60)	NS
Acidemia, *n* (%)	13 (32)	18 (68)	NS
Organ edema, *n* (%)	16 (39)	25 (61)	NS
Mixed, *n* (%)	99 (45)	122 (55)	NS
Comorbidity			
Diabetes, *n* (%)	63 (49)	94 (44)	NS
Hypertension, *n* (%)	90 (70)	135 (64)	NS
Congestive heart failure, *n* (%)	26 (21)	51 (24)	NS
Liver cirrhosis, *n* (%)	9 (7)	38 (18)	.003
Chronic kidney disease, *n* (%)	47 (41)	67 (59)	NS
Ischemic heart disease, *n* (%)	37 (34)	71 (66)	NS
Systolic blood pressure (mmHg)	86 ± 25	79 ± 21	.003
Diastolic blood pressure (mmHg)	52 ± 14	49 ± 13	NS
APACHE 2 score	24.5 ± 5.8	26.9 ± 5.7	<.001
SOFA score	10.3 ± 3.1	12.5 ± 2.9	<.001
RBC transfusion	2.7 ± 3.2	2.9 ± 4.6	NS
Hemoglobin (mg/dl)	10.3 ± 2.4	10.4 ± 2.4	NS
Serum creatinine (mg/dl)	4.2 ± 2.8	3.3 ± 2.7	.007
Total bilirubin (mg/dl)	1.0 ± 1.4	2.7 ± 6.1	.002
Serum albumin (mg/dl)	3.0 ± 0.7	2.8 ± 0.6	.001
WBC count (× 10^3^/mL)	15.3 ± 8.9	14.9 ± 9.2	NS
C-reactive protein (mg/dl)	11.6 ± 11.0	11.3 ± 11.2	NS
RDW (%)	14.9 ± 2.1	16.1 ± 3.3	<.001
Electrolyte			
Na (mmol/L)	136 ± 14	138 ± 6	NS
K (mmol/L)	4.5 ± 2.1	4.3 ± 0.9	NS
Cl (mmol/L)	101 ± 9	101 ± 7	NS
Ca (mg/dl)	7.8 ± 1.0	8.0 ± 0.9	NS
P (mg/dl)	5.1 ± 3.1	5.0 ± 2.5	NS
Mg (mmol/L)	2.4 ± 0.7	2.4 ± 0.6	NS
ABGA			
pH	7.29 ± 0.14	7.27 ± 0.16	NS
PaO2 (mmHg)	97.7 ± 36.9	97.2 ± 56.2	NS
PaCO2 (mmHg)	32.2 ± 12.7	34.2 ± 13.1	NS
HCO3 (mmol/L)	14.2 ± 6.8	14.8 ± 6.8	NS
Lactic acid level^a^ (mmol/L)	7.0 ± 7.6	7.9 ± 7.0	NS
Platelet count (× 10^3^/mL)	178 ± 101	134 ± 84	<.001
UO <0.5 ml/kg/h during D1, *n* (%)	84 (66)	182 (86)	.001

APACHE: Acute Physiology and Chronic Health Evaluation; SOFA: sepsis-related organ failure; RBC: red blood cell; WBC: white blood cell; RDW: red blood cell distribution width; UO: urine output; ABGA: arterial blood gas analysis.

a Lactic acid level was available in 255 patients.

### Predicting survival in septic AKI patients undergoing CRRT

A univariate analysis indicated that age, serum albumin level, RDW value, APACHE II score, platelet count, serum creatinine level, presence of liver cirrhosis, and urine output <0.05 mL/kg/h the first day were significant predictors of mortality in sepsis-induced AKI patients undergoing CRRT. After adjusting for these factors in a multivariate logistic regression analysis, age, APACHE II score, RDW value, platelet count, serum creatinine level, and a urine output <0.05 mL/kg/h the first day were prognostic factors for the 28-day all-cause mortality in sepsis patients needing CRRT (Table 4).

**Table 4.** Predictors of mortality (univariate and multivariate analysis).

	Univariate		Multivariate	
	HR (95%CI)	*p*-value	HR (95%CI)	*p*-value
RDW	1.180 (1.074–1.296)	.001	1.157 (1.043–1.284)	.006
Age	1.028 (1.011–1.045)	.022	1.028 (1.006–1.050)	.013
Albumin	0.567 (0.408–0.789)	.001	0.800 (0.539–1.189)	.270
Transfusion	0.706 (0.958–1.066)	.7	0.976 (0.915–1.041)	.458
APACHE II score	1.073 (1.031–1.116)	<.001	1.054 (1.004–1.106)	.036
Serum creatinine	0.895 (0.821–0.975)	.011	0.862 (0.786–0.946)	.002
Platelet count	0.995 (0.992–0.997)	<.001	0.996 (0.993–0.999)	.013
Presence of LC	2.863 (1.335–6.143)	.007	2.098 (0.815–5.401)	.125
Presence of septic shock	3.249 (2.000–5.289)	<.001	2.980 (1.704–5.213)	.001
UO <0.5 ml/kg/h during the first day	3.178 (1.868–5.405)	<.001	2.710 (1.451–5.061)	.002

APACHE: Acute Physiology and Chronic Health Evaluation; LC: liver cirrhosis; RDW: red blood cell distribution width; UO: urine output.

## Discussion

Sepsis is a major unresolved medical challenge. It is the leading cause of AKI and contributes to multiple organ dysfunction syndrome, a very serious medical condition [5,16]. The CRRT is an important treatment modality for sepsis because it can extract or lower the concentrations of many pro-inflammatory mediators, and therefore has immunomodulatory effects [6,17,18]. Patients with septic AKI have an increased risk for death and a longer average duration of hospitalization [13]. In patients with sepsis-induced AKI undergoing CRRT, the 28-day mortality ranged from 38% to 66% in previous reports [19–22]. The mortality was 62% in our study, comparable to previous reports. This finding might be explained by the similar APACHE II scores (which reflect a patient’s severity) in this and the previous studies.

Most previous studies on CRRT have included both septic and non-septic AKI patients, whereas only septic AKI patients were included in the present study. Age, RDW value, APACHE II score, platelet count, serum creatinine level, and urine output the first day of CRRT were predictors for the 28-day all-cause mortality in sepsis patients undergoing CRRT in this study. The RDW, routinely reported as part of a complete blood cell count, is a simple laboratory test that is used to evaluate variability in the size and form of red blood cells [23,24]. Several studies have shown that an elevated RDW value is a predictor of morbidity and mortality in cardiovascular diseases [25,26]. Recently, it was reported that a high RDW value is also associated with mortality in older patients with sepsis and in patients treated with CRRT [27,28]. Our study revealed that the RDW score can be a prognostic factor predicting mortality in sepsis-induced AKI patients undergoing CRRT. Although the mechanism underlying the association between higher RDW and adverse outcome in patients with sepsis is not completely understood, several mechanisms can be postulated. First, systemic inflammation response in sepsis inhibits erythrocyte maturation and accelerates the migration of reticulocytes into the peripheral circulation, thereby increasing RDW [29]. Second, high oxidative stress in sepsis, which is present through the generation of reactive oxygen species by activated leukocytes, induces an increasing RDW by reducing red blood cell (RBC) survival and increasing the release of large premature RBCs into the peripheral circulation [30,31]. In addition, the release of cytokine in response to inflammatory stress might block the activity of erythropoietin and inhibit erythrocyte maturation and cause production of ineffective red blood cell and elevated RDW [32]. To our knowledge, this is the first report to show an association between RDW and mortality in patients with sepsis-induced AKI undergoing CRRT. Therefore, when starting CRRT for sepsis-induced AKI patients with a high RDW score, it may be necessary to treat aggressively and carefully.

The APACHE II score is a prognostic factor in both sepsis and AKI patients [33,34]. In our study, the APACHE II score was also a risk factor to predict mortality in patients with sepsis-induced AKI undergoing CRRT. The APACHE II score was higher in non-survivors than survivors (24.57 ± 5.89 vs. 26.92 ± 5.76, *p* < .01). It is also an authoritative scoring system for assessing the severity of critical illness, which is also shown in our study. The non-survivors had more frequency of shock than those of survivor. In addition, old age is a well-known predictor of mortality in sepsis patients and in patients receiving CRRT [35,36]. This finding was confirmed in our study in that the survivors were younger than the non-survivors (64 ± 14 years vs. 69 ± 12 years, *p* < .01). Thrombocytopenia is common in patients receiving CRRT [32,37]. Wu et al. reported that a low platelet count may be associated with hospital mortality in patients treated by CRRT [38]. In the present study, the average platelet count was higher in the survivors than in the non-survivors (178 ± 101 × 10^3^/mL vs. 134 ± 84 × 10^3^/mL, *p* < .01), and a low platelet count was a risk factor to predict mortality. Therefore, in patients with sepsis-induced AKI undergoing CRRT, caution is recommended when treating older patients and those with a high APACHE II score or a low platelet count.

There are some reports that levels of creatinine at the time of CRRT initiation do not have a negative influence on mortality [39]. Other reports suggest that high creatinine levels at the time of CRRT initiation are associated with better patient survival [40,41]. The latter finding was supported by our study. Serum creatinine levels are dependent upon age, gender, nutritional status, presence of liver disease, and fluid volume status [42,43]. Non-survivors had more liver cirrhosis than survivors in our study. It is possible that non-survivors also had lower serum creatinine levels than survivors in our study because of decreased production due to liver disease and old age in the non-survivor group. Although serum creatinine was not identified as a risk factor, urine output on CRRT initiation did have an important impact in our study [44,45], which was also shown in our study.

Several literatures reported that there is no significant difference in mortality between patients who receive continuous and intermittent renal replacement therapies [46,47]. The Surviving Sepsis Campaign guidelines suggest using CRRT to facilitate of fluid balance in hemodynamically unstable septic patient [47]. In our study, the majority of patients are hemodynamically unstable at the time of initiation of RRT. Thus, we chose the CRRT as a RRT in treating septic AKI patients. However, there is no strong evidence that CRRT is superior to intermittent hemodialysis for AKI in septic shock patients. We believe that more studies are needed to clarify this.

Our study had certain limitations. First, this was a retrospective study, and the study population comprised only Asian participants. Second, the number of patients was relatively small. Thus, a large, prospective, randomized, controlled study is needed. Third, our database only contained data about patients who underwent CVVHDF. Fourth, we cannot measure body weight in 20 patients in ICU before starting CRRT because of patient’s condition and limited number of bed scale. In those cases, we estimated body weight using abdominal and thigh circumference [48].

In our study, the 28-day all-cause mortality in sepsis-induced AKI patients undergoing CRRT was 62%. Age, APACHE II score, RDW score, platelet count, serum creatinine level, and a urine output <0.5 mL/kg/h the first day were predictors for the 28-day all-cause mortality in these patients. Therefore, it will be helpful to consider these factors in the future when treating sepsis-induced AKI patients undergoing CRRT.
